# High Inter‐Specific Diversity and Seasonality of Trunk Radial Growth in Trees Along an Afrotropical Elevational Gradient

**DOI:** 10.1111/pce.15295

**Published:** 2024-11-24

**Authors:** Lenka Plavcová, Jan Tumajer, Jan Altman, Miroslav Svoboda, Annemiek Irene Stegehuis, Vít Pejcha, Jiří Doležal

**Affiliations:** ^1^ Department of Forest Ecology, Faculty of Forestry and Wood Sciences Czech University of Life Sciences Prague Czech Republic; ^2^ Department of Physical Geography and Geoecology, Faculty of Science Charles University Prague Czech Republic; ^3^ Institute of Botany of the Czech Academy of Sciences Průhonice Czech Republic; ^4^ Laboratoire de Géologie, IPSL, CNRS UMR 8538, École Normale Supérieure PSL University Paris France; ^5^ Faculty of Science University of South Bohemia České Budějovice Czech Republic

**Keywords:** dendrometer, forest, growth dynamics, limitation, soil moisture, temperature, tropics

## Abstract

Understanding mechanisms driving tropical tree growth is essential for comprehending carbon sequestration and predicting the future of tropical forests amid rapid deforestation. We conducted a natural experiment in Mount Cameroon to identify climatic factors limiting diurnal and seasonal growth in dominant tree species across a 2200‐m elevation gradient, from lowland rainforests to montane mist forests with distinct wet and dry seasons. Using high‐precision automatic dendrometers, we recorded radial growth rates of 28 tropical tree species from 2015 to 2018, correlating them with rainfall (11 100–2500 mm) and temperatures (23–14°C) across elevations. Significant growth limitations were suggested at both extremes of water availability. Tree growth peaked during the dry and prewet seasons at humid lower elevations and during wet seasons at drier higher elevations. Growth rates increased with soil moisture at higher elevations and peaked at medium soil moisture at lower elevations. Trees grew fastest at lower temperatures relative to their elevation‐specific means, with growth limited by high daytime temperatures and promoted by nighttime temperatures. Our results revealed significant interspecific diurnal and seasonal growth variations hindered by both water scarcity and excess in West African rainforests, essential for forecasting and modelling carbon sinks.

## Summary statement

- This study uses high‐precision automatic dendrometers to explore the seasonality of trunk radial growth in trees growing along a 2200‐m elevation gradient on Mount Cameroon.
- Our results suggest that radial growth was limited by water scarcity at high elevations and water excess at low elevations.

## 1 Introduction

Tropical forests, despite covering less than 10% of the global land area, host 60%–70% of tree species diversity (Cazzolla Gatti et al. 2022), store 25% of the terrestrial above‐ and belowground carbon (Bonan 2008), and contribute to over one‐third of primary productivity (Beer et al. 2010). However, widespread deforestation threatens the biodiversity and ecosystem functions of these sensitive ecosystems (Giam 2017). Understanding the growth of trees in tropical regions is crucial for their restoration and maintenance in the face of rapid environmental change (Seymour and Harris 2019). Despite their significance for carbon sequestration (Lewis et al. 2009; Cuni‐Sanchez et al. 2021) and presumably high climate resilience (Bennett et al. 2021), African forests are less well‐characterised among tropical forests worldwide.

The growth responses of plants are important feedback mechanisms for environmental changes. However, our understanding of growth traits in tropical plants, such as growth rates and seasonality, is still limited (Lewis et al. 2013; Zuidema et al. 2022; Zhou et al. 2023). In temperate forests, annual growth increments can be easily estimated using tree‐ring analyses. However, in tropical regions, trees often do not have distinct tree‐ring boundaries, leading to the assumption that tree growth is continuous throughout the year (Whitmore 1990; Bräuning et al. 2008). Nevertheless, even in relatively stable tropical climates, significant seasonality in rainfall or temperature occurs, which may lead to periods more conducive to growth (Dünisch, Montóia, and Bauch 2003; Bendix et al. 2006; Bräuning et al. 2008). In fact, annual growth periodicity is well‐documented in seasonally dry tropical environments, where trees often form clear annual growth rings due to growth quiescence during the dry season (Fichtler et al. 2004; Trouet, Coppin, and Beeckman 2006). Surprisingly, growth seasonality resulting in annual ring formation has also been observed in ever‐wet tropical forests in the Amazon, with growth depression observed during the wettest months (Giraldo et al. 2020; Giraldo et al. 2023). This evidence suggests that tropical tree growth can be limited on both sides of the water availability gradient; however, more research is needed to understand these limitations better.

High‐precision electronic dendrometers are useful for studying trunk radial variation in both temperate and tropical trees (Worbes 1995; Etzold et al. 2022). These dendrometers continuously record variations in trunk radial size with sub‐hourly resolution, allowing for the assessment of hourly, daily, monthly, or yearly growth rates, as well as the characterisation of tree water storage (De Swaef et al. 2015; Zweifel et al. 2016; Salomón et al. 2022). In recent years, our understanding of temperate tree growth and its drivers has been improved using dendrometer data and the underlying theoretical predictions (De Swaef et al. 2015; Peters et al. 2021). For example, it has been found that temperate trees primarily grow at night when the tree water balance and cell turgor are favourable for cell growth and expansion within the cambial zone (Zweifel et al. 2021). Availability of soil and atmospheric moisture also constrains growth at a seasonal scale, as growth occurs intermittently only on certain days within a broader seasonal window defined by day length and temperature (Etzold et al. 2022; Tumajer et al. 2022). Therefore, tree growth is limited by many factors, and dendrometer studies can help to understand these factors better.

Despite their potential to provide important insights into tree growth dynamics, the use of dendrometers in tropical and subtropical environments remains scarce. In a study of two tropical conifers and two broadleaved species growing in southeastern Ethiopia, which experiences two wet seasons per year, all trees showed clear cambial dormancy during the dry season (Krepkowski et al. 2011). In contrast to the conifers, which could initiate cambial growth during a short wet period, the broadleaved trees grew only during the long wet season (Krepkowski et al. 2011). Xylem and leaf growth occurred primarily during the wet season for trees on two tropical dry forest sites in South America, and the hourly growth rates of those trees were negatively affected by high air temperatures (Mendivelso et al. 2016). More recently, coherent diel and seasonal growth patterns, mainly driven by air temperature and vapour pressure deficit (VPD), have been observed across 14 species growing in a subtropical montane forest (Zhou et al. 2023). These and other dendrometer studies illustrate the seasonal variation of growth in tropical and subtropical environments and point to the main environmental drivers. However, given the great interspecific diversity of tropical forests and the vast variability in environmental conditions, more studies are needed to substantiate these growth‐climate links.

In the present study, we studied the intra‐annual variability of tree growth of 28 different species. These trees were spread across a 2200 m elevational gradient on Mount Cameroon over 4 years (2015–2018). The region experienced rainfall ranging from 11 100 to 2500 mm and temperatures from 23 to 14°C, from the lowest to the highest elevation. We investigated if the diversity in annual and daily trunk radial growth patterns among tropical tree species is influenced by varying temperatures and water availability along the elevational gradient. Despite the limited seasonality typical of tropical climates, we anticipated finding significant seasonal and diurnal patterns in radial tree growth, primarily influenced by moisture availability. We hypothesised that both ends of the water availability spectrum restrict tree growth in tropical climates, indicating that both water scarcity and water excess can hinder growth. We hypothesised that high soil moisture suppresses tree growth during the wet season at perhumid lower elevations with > 11 000 mm rainfall, possibly due to root anoxia and nutrient leaching. At mid‐elevations, tree growth might follow a continuous pattern throughout the year. In contrast, the montane trees at higher elevations would experience reduced growth, water deficit, and stem shrinkage during the dry season.

## 2 Methods

### 2.1 Study Site

**Figure 1:**
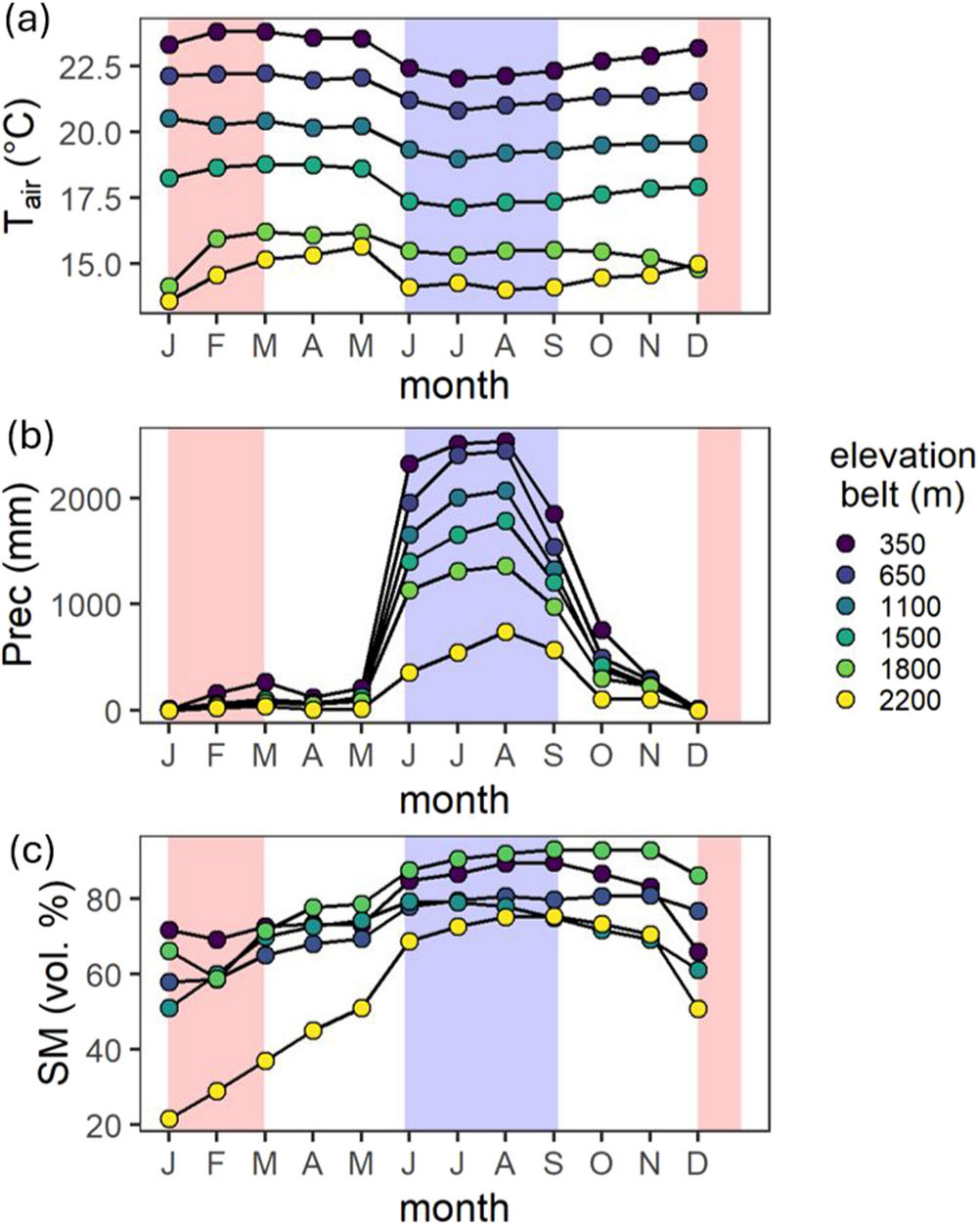
(a) Monthly mean air temperature (Tair, °C), (b) monthly mean precipitation sum (Prec, mm) and (c) monthly mean volumetric soil moisture (SM, vol. %) in six elevation belts along the slope of Mount Cameroon. The wet season is depicted as blue area, while the dry season in depicted in red.

The research was conducted in a tropical forest on the southwest slopes of Mount Cameroon in the Republic of Cameroon (4°07′ N, 9°05′ E). This area features volcanic soils with high permeability and is covered by primary tropical forests up to 2200 m a.s.l., where the vegetation shifts abruptly to the Afromontane savanna (Proctor et al. 2007). Over 100 permanent monitoring plots were established along the slope, categorised into six elevation zones: 350, 650, 1100, 1500, 1800, and 2200 m above sea level (Doležal et al. 2022; Řeháková et al. 2022). The first two zones (350 and 650 m) are lowland rainforests, the middle zones (1100 and 1500 m) are transition forests, and the highest zones (1800 and 2200 m) are montane forests with significant cloud cover and frequent mist. The study area experiences a wet tropical climate influenced by alternating southwest maritime winds and northeast dry continental winds, resulting in distinct seasonal rainfall patterns with a dry season from December to February and a wet season from June to September. As elevation increases, both temperature and precipitation decrease (Figure 1).

### 2.2 Microclimate Conditions

Microclimate conditions for each elevation belt were monitored using loggers that recorded air temperature (*T*_air_), precipitation, and soil moisture. *T*_air_ was recorded using DRL26 loggers (EMS Brno, Czech Republic) attached to tree trunks, while Minikin ERi measured precipitation with Pronamic Pro Rain Gauges (EMS Brno, Czech Republic) placed in regularly cleared microsites. Soil moisture was monitored using TMS‐4 Standard soil moisture sensors and dataloggers (TOMST, Prague, Czech Republic) installed at a depth of 6 cm. These microclimate data have been collected since 2015 and have been previously published (Řeháková et al. 2022; Plavcová et al. 2024). Environmental data availability varies across elevation belts and parameters due to frequent logger damage in the dynamic and harsh tropical environment. *T*_air_ data cover the full 4‐year period (2015–2018) across all elevation belts, while precipitation data vary in length from one to 4 years. Soil moisture data are available for 2015 and 2016.

### 2.3 Measurement of the Stem Radial Variation and Data Processing

In 2015, we installed 60 high‐precision band dendrometers (DRL26C; EMS Brno, Brno, Czech Republic) on selected trees within a subset of the monitoring plots, choosing 10 species per elevation belt to ensure a wide range of species diversity and represent dominant species in both lower and upper canopy strata. The selected trees had regular stem shapes and diameters at breast height (DBH) between 10 and 100 cm (see Table 1). The dendrometers were installed at breast height after removing the outermost dead bark. We recorded dendrometer readings at 30‐min intervals from January 2015 to December 2018 and periodically downloaded the data. The raw data were processed using the “treenetproc” package (Knüsel et al. 2021) in R (R Core Team 2022). Initially, the data were time‐aligned, and any artificial jumps and shifts were removed. We discarded time series with significant missing or erratic data, resulting in a final data set of 30 individual trees (28 species, see Table 1) with readings from over 90% of the growth season for all 4 years (2015‐2018).

**Table 1:** Study species growing in six elevation belts along the slope of Mount Cameroon. Diameter in the breast height (DBH) and tree height and are shown.

Elevation belt (m)	Abbrev.	Species	Family	DBH (cm)	Height (m)
350	DIBI	*Diospyros bipindensis*	Ebenaceae	22.4	13
350	STPU	*Strombosia pustulata*	Olacaceae	23.1	16
350	TAAF	*Tapura africana*	Dichapetalaceae	18.8	16
350	TEDI	*Tetrorchidium didymostemon*	Euphorbiaceae	30.3	24
350	UAST	*Uapaca staudtii*	Phyllanthaceae	47.1	24
350	VIGR	*Vitex grandifolia*	Lamiaceae	29.2	15
650	ANFR	*Anthonotha fragrans*	Fabaceae	47	21
650	DIZE	*Diogoa zenkeri*	Olacaceae	16.8	12
650	ZAGI	*Zanthoxylum gilletii*	Rutaceae	33.6	21
1100	HOLE	*Homalium letestui*	Salicaceae	13	10
1100	POSU	*Polyalthia suaveolens*	Annonaceae	33	23
1100	SCMA	*Schumanniophyton magnificum*	Rubiaceae	19.2	7
1100	TEAF	*Teclea afzelii*	Rutaceae	21.5	13
1500	ENUT	*Entandrophragma utile*	Meliaceae	83.5	35
1500	MAOC	*Macaranga occidentalis*	Euphorbiaceae	25.95	17
1500	ONLO	*Oncoba lophocarpa*	Achariaceae	43.4	13
1500	SYST	*Syzygium staudtii*	Myrtaceae	17.55	14
1500	TACR	*Tabernaemontana crassa*	Apocynaceae	17.1	12
1500	XYST	*Xylopia staudtii*	Annonaceae	38.7	22
1500	ZAGI	*Zanthoxylum gilletii*	Rutaceae	31.4	15
1800	ILMI	*Ilex mitis*	Aquifoliaceae	97.1	25
1800	PSDU	*Psydrax dunlapii*	Rubiaceae	19.1	10
1800	SCAB	*Schefflera abyssinica*	Araliaceae	20.5	29
2200	AGSA	*Agauria salicifolia*	Ericaceae	44	20
2200	CLAN	*Clausena anisata*	Rutaceae	15.8	8
2200	MYAR	*Myrica arborea*	Myricaceae	32	8
2200	NUCO	*Nuxia congesta*	Stilbaceae	10.4	7
2200	OLCA	*Olea capensis*	Oleaceae	31.3	18
2200	RAME	*Rapanea melanophloeos*	Myrsinaceae	21.3	17
2200	SYST	*Syzygium staudtii*	Myrtaceae	25.4	17

The collected data were converted from circumference growth to radius growth by dividing the circumference by 2*π, assuming a circular stem profile. For each species, the data were subsampled to hourly, daily, weekly, monthly, and yearly scales for further analysis. The data were decomposed into irreversible trunk radial growth and reversible shrinking and swelling due to changing water content inside the trunk. The separation of irreversible growth was carried out using the “zero‐growth” approach of Zweifel et al. (2016), which assumes that no growth occurs until the change in stem radius exceeds the previous maximum. To calculate daily growth rates across the growth year, irreversible growth data (daily GRO) were smoothed by a 2‐week moving average rolling in 1‐day intervals, and the daily growth rate (daily GR) was then calculated by subtracting the two consecutive growth readings. This calculation was done separately for each species and year. From these data, the maximal daily growth rate was calculated as the 90th percentile of all daily GR. Furthermore, we determined the number of growth days within each year by flagging each hour in hourly data as ‘growth’ or ‘no growth’ hour and then filtering all days with at least one growth hour (growth days). Finally, we calculated the annual cumulative growth (annual GRO, mm) per species and year by subtracting the cumulative GRO at the end and at the beginning of each year. We calculated two‐way ANOVA and Tukey's HSD post‐hoc test to identify significant differences in annual GRO, daily maximum GR, and annual number of growth days between elevation belts and years.

We studied the daily shrinking and swelling of tree trunks. To track the daily variation in trunk circumference, we first removed the larger seasonal changes by subtracting a 2‐week moving average from the hourly dendrometer readings. Then, for each day, we subtracted the first reading of the day (hour = 0) from all subsequent readings within that day (Drew and Downes 2009; Jupa et al. 2022). The resulting values were averaged by species and month. We calculated the maximum daily trunk shrinkage (MDS) for each species and month by subtracting the daily maximum and minimum values.

### 2.4 Climatic Drivers of Radial Growth

We conducted a correlation analysis to examine how the radial growth rate was affected by microclimatic conditions. Specifically, we looked at the relationship between hourly growth rate (hourly GR) and air temperature as well as soil moisture, which microclimatic loggers recorded at the specific site. We used Pearson correlations to analyse the data for individual plant species and then aggregated the results for the different elevation levels. To check for the consistency of results across a wider time interval, this analysis was additionally done for growth rate data on daily and weekly scales (i.e., daily GR and weekly GR).

To understand the variation in the climate‐growth responses throughout the year and day, we calculated correlation coefficients separately for each calendar month and hour of the day. Additionally, we categorised air temperature and soil moisture into intervals of 2°C and 10% (v/v), respectively. We then calculated the average value of hourly GR for each interval to visualise how the growth rate responds to climatic conditions for different plant species and elevation levels. This analysis was also carried out for daily GR and weekly GR.

Finally, we developed a linear mixed‐effect model using the ‘lme4’ package (Bates et al. 2014) in R (R Core Team 2022) to quantify the levels of diurnal, seasonal, and interspecific variability in the growth rate and its responses to climate. The model used hourly GR as the response variable and temperature and soil moisture as fixed predictors. The random component of the model allowed for variation in the mean hourly GR (i.e., intercept) and the responses to climatic variables (i.e., slopes) between different hours of the day, months of the year, and plant species within each elevation level. We then compared the proportions of variability accounted for by each of these random predictors.

## 3 Results

### 3.1 Site Microclimatic Conditions

Along the elevation gradient, both air temperature (*T*_air_) and precipitation (Prec) decreased with increasing elevation (see Figure 1a,b). The mean annual temperature for the 350‐metre elevation was 23°C and decreased gradually to 14.6°C for the 2200‐metre elevation. Annual precipitation was 11 070 mm at 350 m and decreased to 2521 mm (a decrease of 77%) at 2200 m. Air temperature showed only a weak seasonal pattern, with the wet season being approximately 1.5°C colder than the dry season. In contrast, precipitation exhibited strong seasonality, with most precipitation (83%–88%) occurring between June and September. Soil moisture reflected the rain seasonality and was lowest at the highest elevation (2200 m), while the differences between the other elevation belts were less distinct (see Figure 1c).

### 3.2 Trunk Radial Growth and Daily Growth Rate Seasonal Dynamics

**Figure 2:**
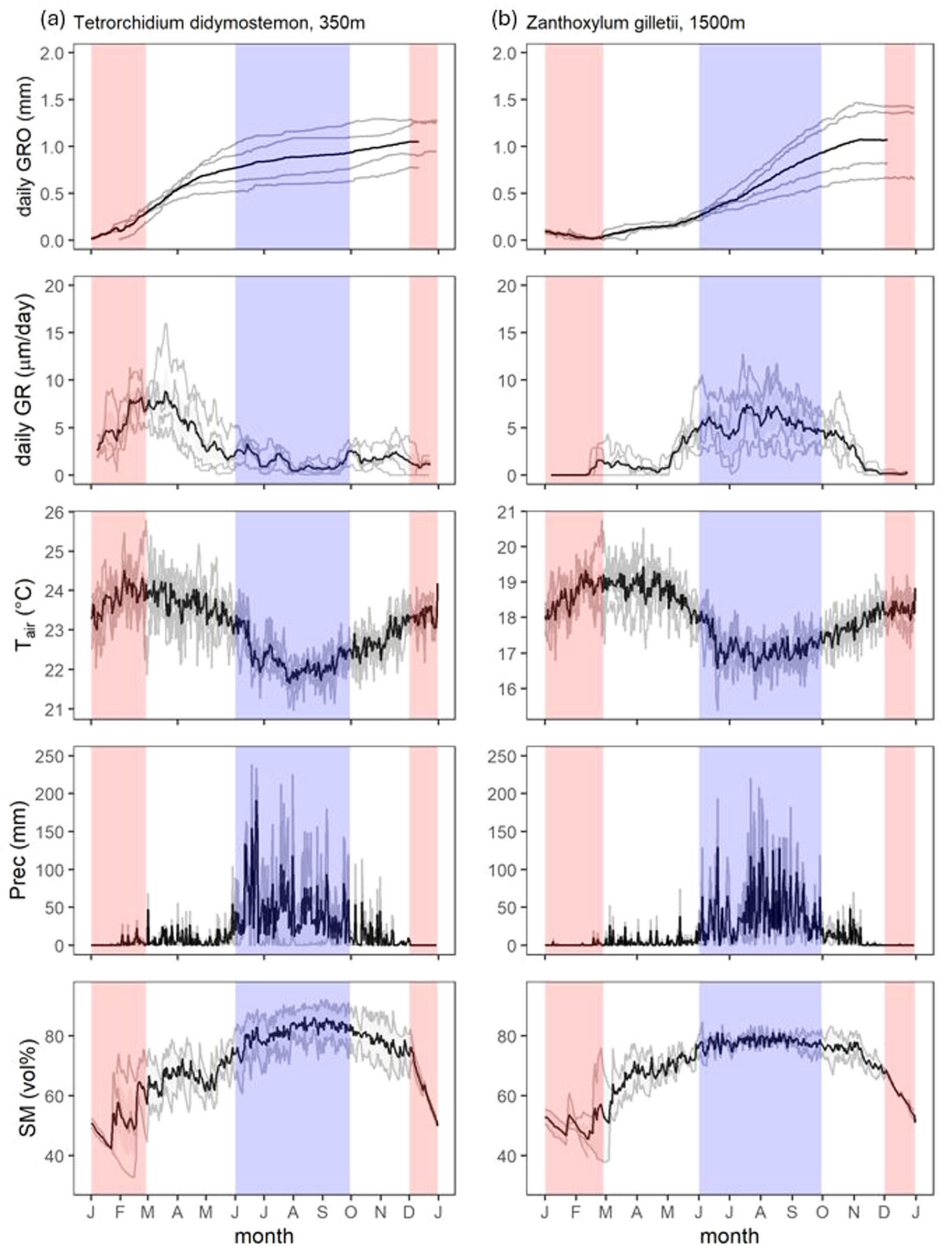
Daily trunk radial growth (daily GRO, mm) and daily growth rate (daily GR, µm/day) for (a) *Tetrorchidium didymostemon* (Euphorbiaceae, growing in a lower elevation of 350 m) and (b) *Zanthoxylum gilletii* (Rutaceae, growing in a higher elevation of 1500 m). Air temperature (*T*_air_, °C), daily precipitation (Prec, mm) and soil moisture (SM, vol. %) measured for each elevation belt (350 m or 1500 m). Note the different scale for *T*_air_. The grey lines represent measurement from different years and the black line show the average across the multiple years. The wet season is depicted as blue area, while the dry season in depicted in red. [Color figure can be viewed at wileyonlinelibrary.com]

**Figure 3:**
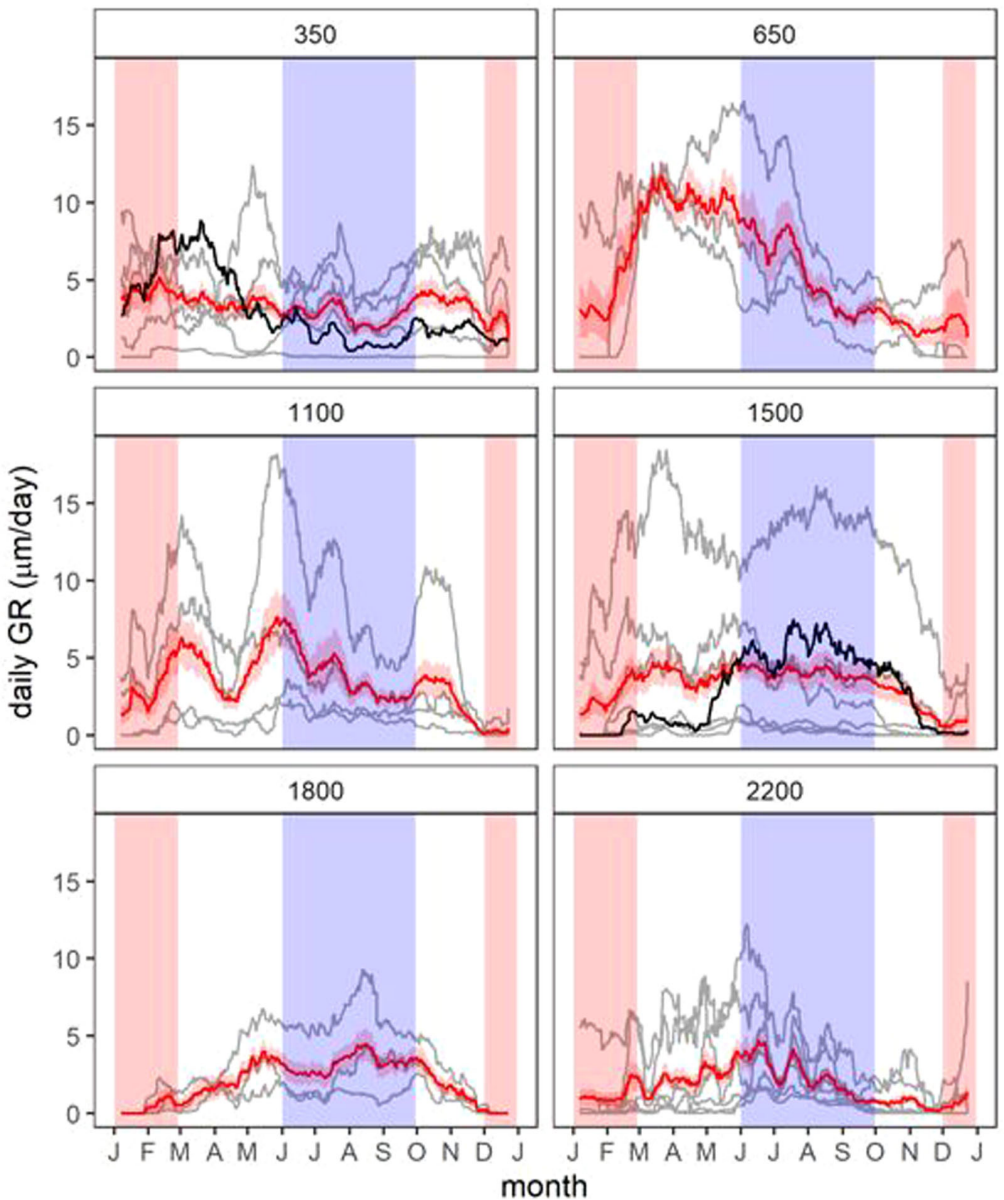
Mean daily growth rates (daily GR, µm/day; mean of the four years of the measurement for each tree) of individual trees (grey lines) and a belt‐level average (red line). The elevation in m a.s.l. is showed in the panel title. The wet season is depicted as blue area, while the dry season in depicted in red. The two lines in black highlight the two species shown in Figure 2 as examples of two contrasting growth patterns. [Color figure can be viewed at wileyonlinelibrary.com]

Daily trunk radial growth (daily GRO) and corresponding daily growth rates (daily GR) displayed distinct seasonal patterns among different species and elevation belts (refer to Figures 2 and 3, Figures S1, S2, S3). The tree growth was mostly linear throughout the year at the lowest elevation belt (350 m a.s.l.). At 650 m a.s.l. and partially also at 1100 m a.s.l., there was a tendency for substantial stem radial growth during the dry season (January–February) and the fastest growth in the prewet period (March–May). In contrast, growth tended to be fastest either in the prewet period (March–May) or in the wet period (June–September) for many tree species growing in mid and high elevations (1500, 1800, 2200 m a.s.l.). The different growth patterns with regard to water availability are illustrated by *Tetrorchidium didymostemon* from the 350 m a.s.l. belt and *Zanthoxylum gilletii* from the 1500 m a.s.l. belt (Figure 2).

### 3.3 Annual Growth Rate, Maximal Daily Growth Rates and Number of Growth Days

**Figure 4:**
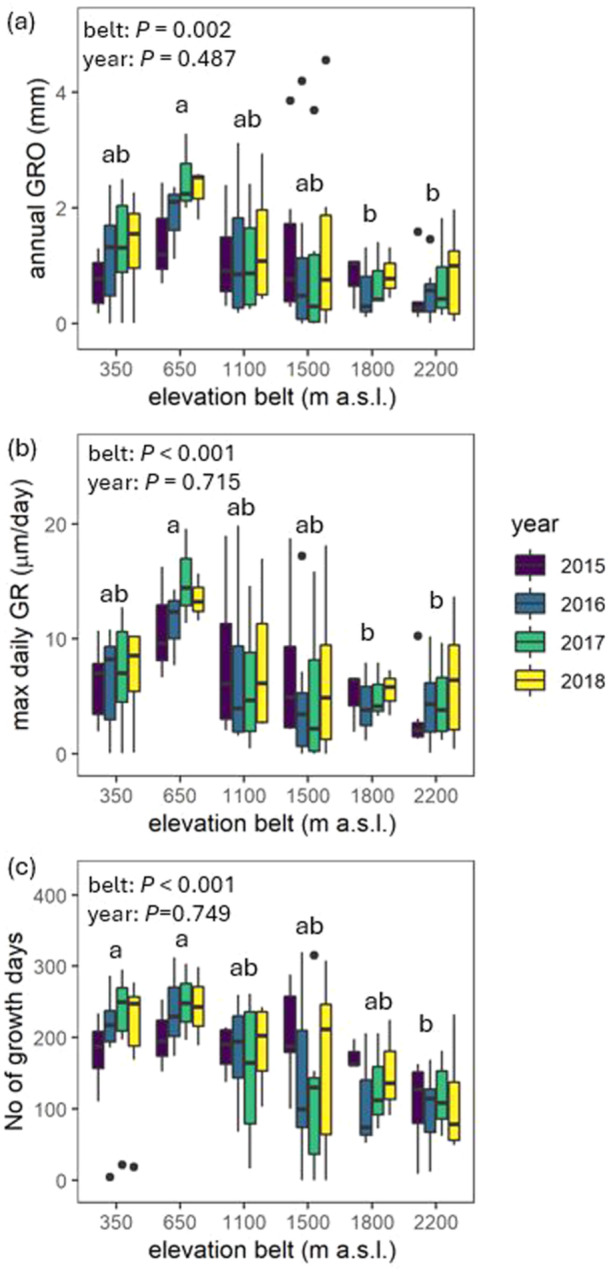
Annual growth (annual GRO, mm) (a), maximal daily growth rate (max daily GR, µm/day) (b) and number of growth days (c) for trees growing in six elevation belts along the slope of Mount Cameroon. Two‐way ANOVA was used for statistical testing of variation in mean values of response variables between belts and years (*p*‐values at the top‐right corner). The different letters indicate statistically significant (*p* < 0.05) differences among elevation belts according to Tukey's post‐hoc test. [Color figure can be viewed at wileyonlinelibrary.com]

The average annual growth (annual GRO) was 1.1 mm across different species and years. It varied from close to 0 in species like *Vitex grandifolia* or *Entandrophragma utile* to 4.6 mm in *Macaranga occidentalis*. Annual GRO was highest for species growing at 650 m above sea level and decreased at higher elevations (see Figure 4a). The maximum daily growth rates (max daily GR) followed a similar pattern and were highest at 650 m above sea level (see Figure 4b). The number of growth days was highest at 350 and 650 m above sea level, reaching 198 and 233 days per year, respectively (see Figure 4c). In contrast, the lowest number of growth days, 108 days per year, was found at 2200 m above sea level (see Figure 4c). Significant differences in annual GRO, number of growth days per year, and maximal daily growth rates occurred between elevation belts but not between years. Mean values of all three variables significantly differed between low and high elevations, typically between 650 and 1800/2200 m a.s.l.

### 3.4 Relationships Between Hourly Growth Rates and Microclimatic Parameters

**Figure 5:**
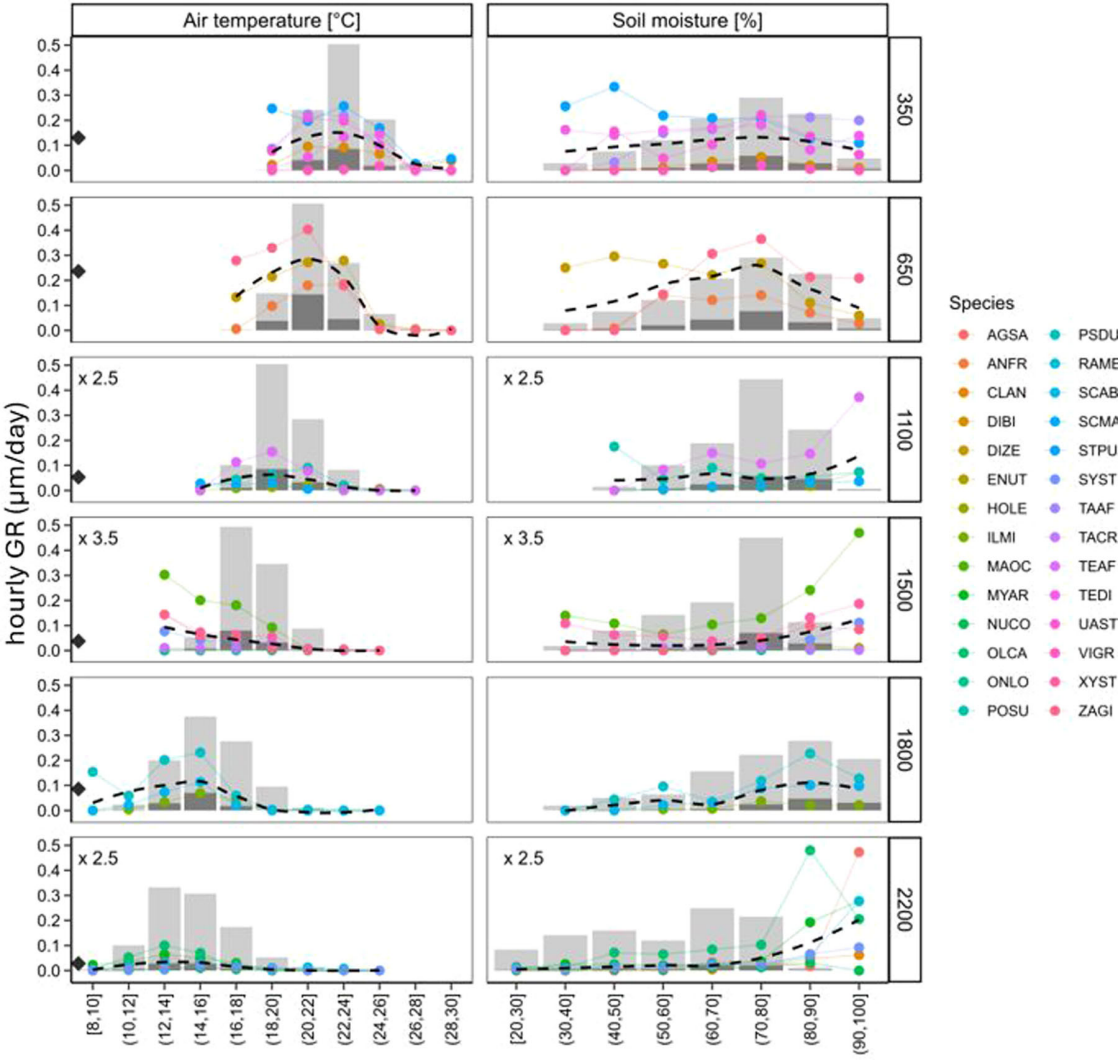
Hourly growth rates (hourly GR, µm/hour) across the range of air temperature and soil moisture for trees growing in six elevation belts along the slope of Mount Cameroon. The points and lines represent individual trees and the belt mean is indicated by the dashed line. Grey bars indicate relative frequency of climatic conditions across different ranges in given elevation belt. Bars are divided into darker and lighter parts to indicate the relative frequency of hours when hourly GR > 0 (darker) and hourly GR = 0 (lighter). Diamond next to *y* axes highlights long‐term mean hourly growth rate for given elevation belt. Note that growth rates of trees from belts 1100 and 2200 m a.s.l. were divided by 2.5 and growth of trees from 1500 m a.s.l. was divided by 3.5 to aid comparison between individual panels on the same scale of *y* axes. [Color figure can be viewed at wileyonlinelibrary.com]

At an hourly scale, the trees at different elevations grew faster when the temperatures were lower compared to the average temperatures at that elevation. The ideal temperature for growth decreased from 23°C at 350 m above sea level to 13°C at 2200 m above sea level (see Figure 5). In mid and high elevation areas, the trees showed the highest hourly GR in wet soil (soil moisture > 80% v/v). In lower elevation sites, there was a weak association between hourly GR and soil moisture, with a possible growth peak at medium soil moisture (70%–80% v/v) and a slight decline in both higher and wetter soils (see Figure 5). The results were similar when the growth rates were considered on a daily and weekly scale (Figure S4 and S5), except that the fastest GR were observed at high temperatures in low elevations.

**Figure 6:**
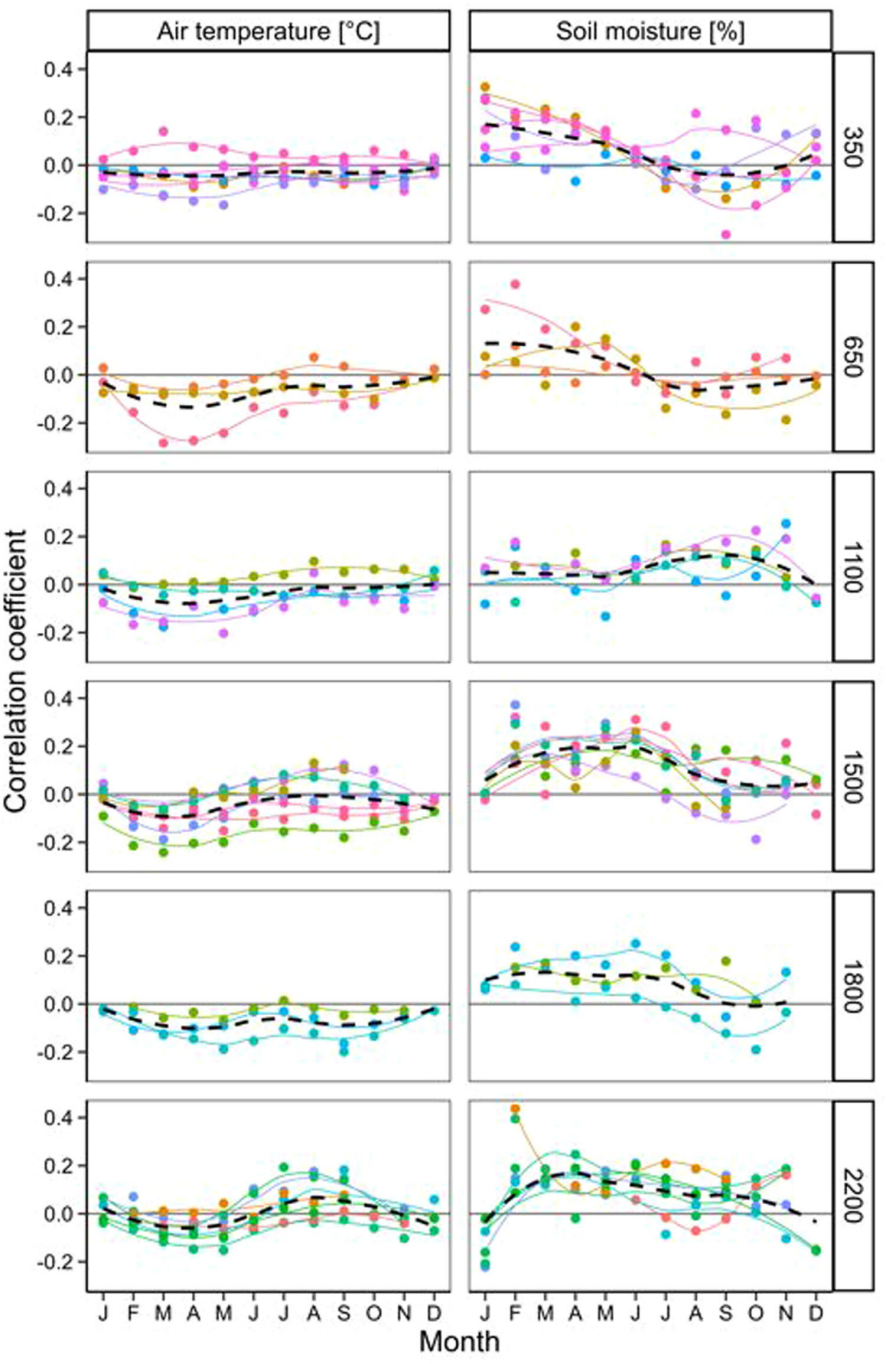
Correlation coefficients between climatic variable (air temperature and soil mositure) and hourly growth rates (hourly GR) during specific calendar months for trees growing in six elevation belts along the slope of Mount Cameroon. The points and lines represent individual trees and the belt mean is indicated by the dashed line. For colour legend refer to Figure 5. [Color figure can be viewed at wileyonlinelibrary.com]

The average correlation coefficient between hourly GR and air temperature was weakly negative across all months (see Figure 6), with almost no seasonal variation except for the highest elevation, where it was weakly positive during the late wet season months. The average correlation coefficient between hourly GR and soil moisture was positive for higher elevation sites, while in lower elevation sites (350, 650 m a.s.l), it was negative during the wet and post‐wet periods (see Figure 6). The correlation coefficients showed similar patterns when data were considered on daily and weekly scales (Figure S6, S7).

On a sub‐daily scale, temperature‐growth correlations showed significant daily variation (see Figure S8). For all elevation belts and most species, growth rates responded negatively to high temperatures during the day but positively at night. In contrast, the correlations between growth rates and soil moisture were less variable over the course of the day, with a slight peak around mid‐day and a minimum after midnight.

A linear mixed‐effect model showed that seasonal, diurnal, and interspecific variability were of similar magnitude in the case of the mean values of hourly GR and its sensitivity to air temperature (see Figure S9). However, there was negligible interspecific variation in a response of hourly GR to soil moisture availability.

### 3.5 Diurnal Trunk Shrinking and Swelling

**Figure 7:**
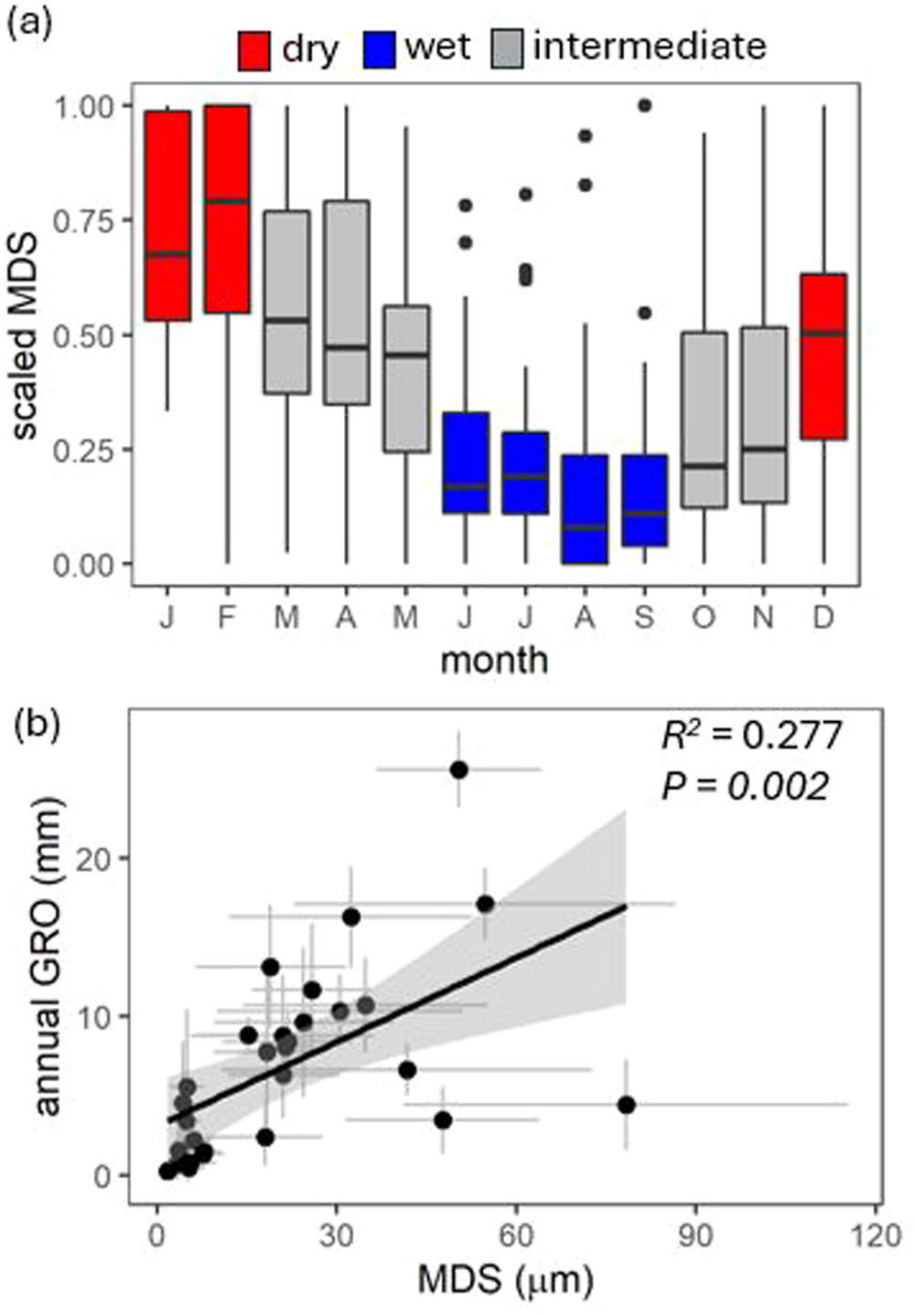
(a) Maximum daily trunk shrinkage (MDS, scaled between 0 and 1) across months of the year. The box in each boxplot indicates 25th and 75th percentiles with the median (central bar). Whiskers represent the 10th and 90th percentiles; outliers are shown as circles. *n* = 30 species, the data for the six elevation belts were pooled. Colours highlight dry (red), wet (blue), or intermediate (grey) season. (b) Relationship between MDS and trunk annual growth (annual GRO, mm). *n* = 30 trees, the data for the six elevation belts were pooled. [Color figure can be viewed at wileyonlinelibrary.com]

The shrinking and swelling of tree trunks throughout the day varied greatly on a day‐to‐day basis, but a clear pattern emerged from the average data for each month and tree species (see Figure 7a and Figure S8). Generally, the trunks swelled from late afternoon to mid‐morning, reaching their maximum size around 8–9 a.m. (Figure S10). After that, they shrank, reaching their smallest size in the late afternoon at around 4–5 p.m. The maximum daily trunk shrinkage (MDS) did not show differences across different elevation levels (Figure S11). However, there was a clear seasonal pattern for MDS, with the highest values occurring during the dry months (December–February) and the lowest values during the wet months (June–September) (Figure 7a, Supporting Information S1: Figure S10,S11). Additionally, MDS was found to be positively correlated with annual GRO (*R* ^2^ = 0.277, *p* = 0.002; Figure 7b) across all tree species.

## 4 Discussion

The elevational gradient on the southwestern slope of Mount Cameroon results in decreasing temperature and precipitation, with a dry season from December to February and a wet season from June to September (Figure 1). This gradient influences the radial trunk growth of the tropical tree species studied, affecting both overall growth rates and seasonality. Annual growth was highest in tropical trees from the lower elevation rainforests (350 and 650 m a.s.l.), where precipitation was most abundant. These trees typically exhibited peak growth during the prewet period (March‐May), which declined or completely ceased during the wet season (June‐September). However, some species maintained consistent continuous growth throughout the year due to high moisture levels. At higher elevations, ranging from 1000 to 2200 m, growth rates were generally lower and occurred primarily during the peak wet season (June–September), with a decline or complete cessation during the dry season (December–February). Our results highlight significant seasonal and species‐specific variability in tree radial growth along the Afrotropical elevational gradient. This was confirmed by the linear mixed‐effects model, which provided numerical estimates of temporal and interspecific growth variation. The diverse growth strategies of the studied trees, evidenced by substantial interspecific variability in daily growth rates and varying seasonality of growth peaks (Figure 3), may contribute to species coexistence in the forest ecosystems on Mount Cameroon. These ecosystems, recognised as major biodiversity hotspots, represent some of the last remaining intact primary tropical forests in West Africa (Proctor et al. 2007; Ferenc et al. 2016; Hořák et al. 2019).

At various elevation zones, the fastest hourly growth occurred at colder temperatures, regardless of the month, except at the highest elevations (Figures 5, 6). It is likely that this pattern is due to comparable magnitude of diurnal and seasonal variation in temperature observed across all elevational belts. On a sub‐daily scale, the negative correlation between radial growth rate and air temperature was highest during daytime hours (Supporting Information S1: Figure S8). This suggests that lower daily temperatures are likely linked to lower evaporative demands, resulting in a positive water balance in the cambial zone, which later promotes cell expansion and division. Recent empirical (Zweifel et al. 2021; Tumajer et al. 2022; Zhou et al. 2023) and modelling studies (Steppe et al. 2006; Peters et al. 2021, 2023) have highlighted the significant influence of vapour pressure deficit and cambial turgor on radial trunk growth. While high temperatures negatively affect radial growth during the day, nighttime cambial activity is unaffected or weakly stimulated by temperature. This is because cambial activity mainly occurs at night when trunk water reserves are replenished (Zweifel et al. 2021), and higher temperatures might only promote cambial activity only at night without increasing transpiration and stem shrinkage. On daily and weekly scales, the trees grew fastest at higher temperatures at least in lower elevations that were presumably less affected by drought (Supporting Information S1: Figures S5, S6). This agrees with observation from temperate and boreal environments, where growth is also usually positively correlated with temperature at a seasonal scale (Rossi, Girard, and Morin 2014; Etzold et al. 2022).

In higher‐elevation montane forests, trees exhibited the fastest growth in soil with high moisture levels, whereas in lower‐elevation rainforests, growth rates peaked in moderately wet soil (Figures 5, 6). The relationship between soil moisture and hourly growth rates highlights the environmental influences on trunk radial growth. The correlation coefficients between growth and soil moisture were consistently positive throughout the year at mid and higher elevations. In contrast, these relationships were negative at lower elevations during the wettest months and at night. These findings suggest that trunk radial growth was limited by drought at higher elevations, consistent with the transition from closed montane forests to open Afromontane savannas (Proctor et al. 2007; Řeháková et al. 2022). It is possible that additional factors, such as the hydraulic properties of highly permeable volcanic soils, abundant horizonal precipitation (i.e. mist), and frequent fires further shape the growth of these high‐elevation trees. Conversely, radial tree growth at lower elevations appeared constrained by excessive moisture. The observation that high soil moisture can hinder tree growth aligns with findings from the ever‐wet Amazonian forest (Giraldo et al. 2023) and temperate forests (Zweifel et al. 2021), where some tree species thrived in moderately moist soil. High soil moisture can create anoxic conditions for roots (Joly and Crawford 1982) and lead to nutrient leaching from the soil, causing nutrient limitation (Campo, Jaramillo, and Maass 1998). Additionally, the reduction in growth during the wet season may be linked to increased cloud cover, which limits photosynthesis due to reduced light availability (Restrepo‐Coupe et al. 2013; Uribe, Sierra, and Dukes 2021). While our data suggest growth limitations at both extremes of water availability, more research that will consider the complex ecohydrology of this unique elevational gradient is needed to confirm this conclusion.

Dendrometers not only record irreversible growth but also provide insights into the daily cyclesof trunk shrinking and swelling, primarily driven by water fluctuations in the bark and xylem (Zweifel, Item, and Häsler 2000; Fernández and Cuevas 2010). On a daily scale, we observed trunk swelling during the night, peaking between 8 and 9 in the morning, while trunks shrank during the day, reaching their minimum at 3–4 in the afternoon. The magnitude of the maximum daily trunk shrinkage (MDS) varied throughout the season, being greatest in the dry months (December–February). This pattern, as well as the magnitude of MDS, was consistent across different elevational belts. The MDS is known to be positively responsive to air temperature and vapour pressure deficit (Fernández and Cuevas 2010; King et al. 2013; Tian et al. 2017; Jupa et al. 2022). These results suggest that trees lose more water via transpiration and rely more on their water storage during the dry season. Across species, MDS was positively correlated with annual growth, indicating that ample water storage can likely support higher transpiration and faster radial growth.

Besides environmental drivers (i.e., temperature and soil moisture) studied here, trunk radial growth is affected by a wide range of other factors, including availability of nutrients (Manu et al. 2022), competition with other trees (Coomes and Allen 2007) and interactions with pests and pathogens (Spear, Coley, and Kursar 2015). In addition, the radial growth is modulated by species‐specific structural and physiological traits such as rooting depth (Kühnhammer et al. 2023), vulnerability to embolism (Chitra‐Tarak et al. 2021), hydraulic efficiency (Santiago et al. 2018) and stomatal regulation (Meinzer et al. 1997). Future studies should establish links between species‐specific growth patterns and water use strategies in these diverse tropical forests.

In conclusion, this study presents community‐wide data on trunk radial growth in a primary tropical forest along a steep environmental gradient. Despite the tropical climate with limited seasonality, we discovered significant seasonal and diurnal patterns in radial growth rates, primarily reflecting moisture availability and/or temperature fluctuations. Additionally, we observed diverse species‐specific annual and daily trunk radial growth patterns, providing insights into potential climatic drivers. On an annual scale, we identified linear growth patterns at low elevations and seasonal growth variation with a peak in the prewet or wet season at high elevations. Growth was observed to occur at lower temperatures, likely due to reduced evaporative demands. The effect of soil moisture was positive at mid‐ and high‐elevation sites, while at lower elevations, growth was suppressed by high soil moisture, likely due to root anoxia and nutrient leaching. Our results indicate growth limitations on both extremes of the water availability spectrum, even in a tropical climate that is not extreme (i.e., neither a seasonally dry tropical forest nor inundated floodplains). Given the seasonal nature of trunk radial growth observed in many species, it would be interesting to determine if the trees formed visible tree rings, offering potential for future dendrochronological studies in this area. In any case, the seasonal nature of radial tree growth and the high inter‐specific variability documented by this study should be considered when forecasting or modelling carbon sinks in tropical forests.

## Conflicts of Interest

The authors declare no conflicts of interest.

## Supporting information

Supporting information.

## Data Availability

The data that support the findings of this study are available from the corresponding author upon reasonable request.
