# Supplementary material for: High Inter‐Specific Diversity and Seasonality of Trunk Radial Growth in Trees Along an Afrotropical Elevational Gradient
Source: Plant Cell Environ. 2024 Nov 24;48(3):2285–97. doi: 10.1111/pce.15295 (PMC11788962; doi:10.1111/pce.15295)
Supplement: Supplementary file 1 — Supporting information. [file PCE-48-2285-s001.docx]

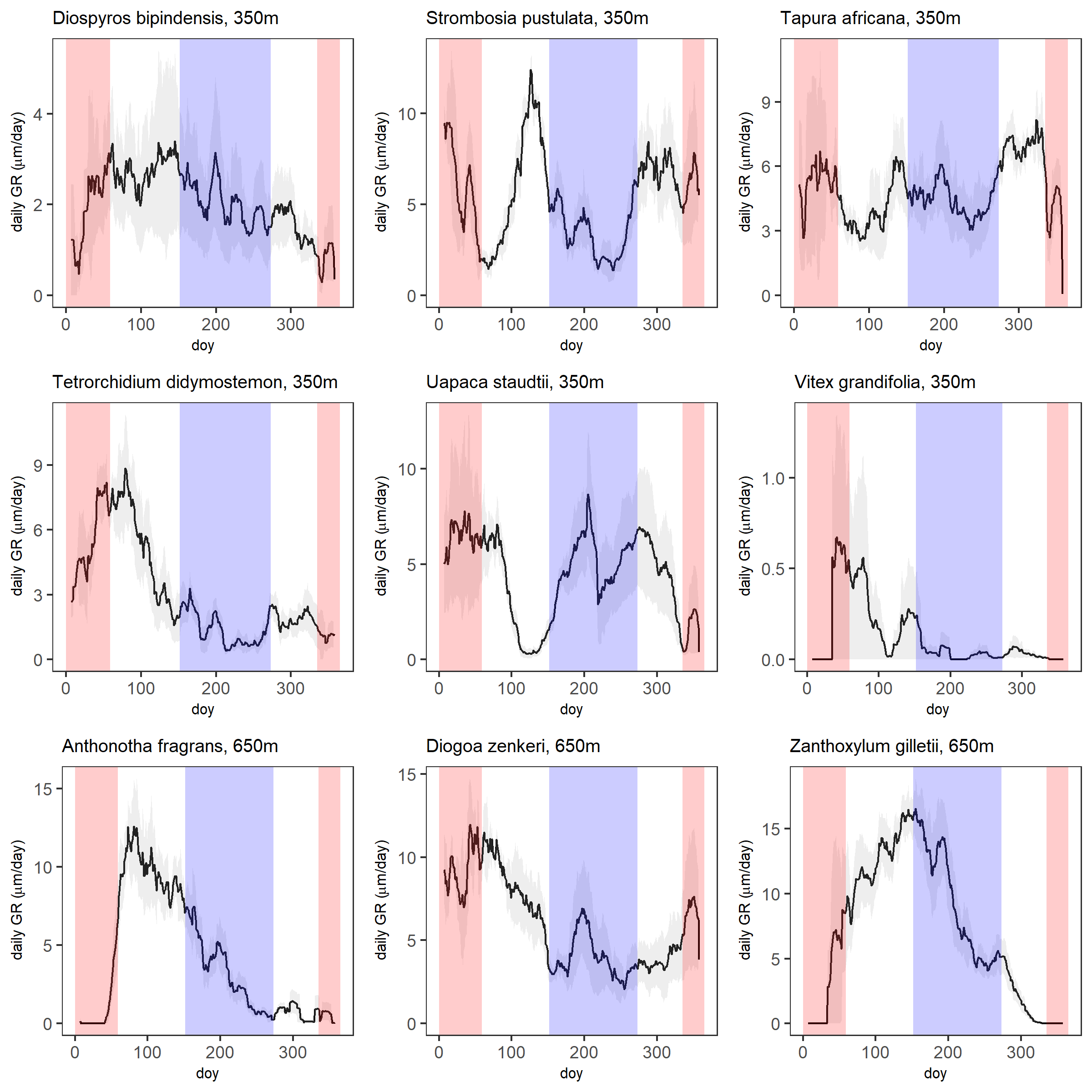


**Figure S1:** Examples of daily growth rate (daily GR) dynamics for selected trees growing in lower elevation s (350, 650 m a.s.l.). Wet season is depicted as blue area, while dry season in depicted in red.


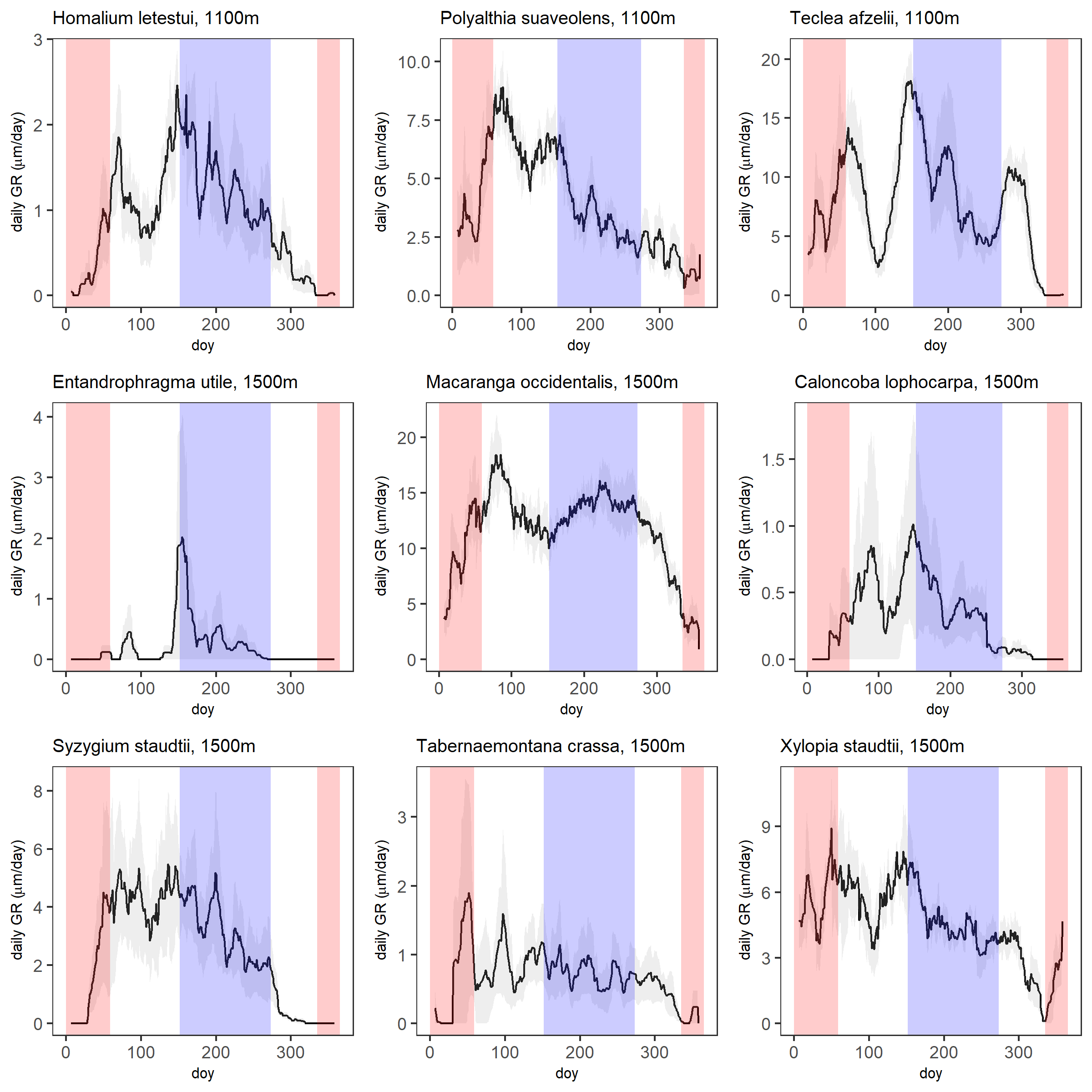


**Figure S2:** Examples of daily growth rate (daily GR) dynamics for selected trees growing in mid elevations (1100 and1500 m a.s.l.). Wet season is depicted as blue area, while dry season in depicted in red.


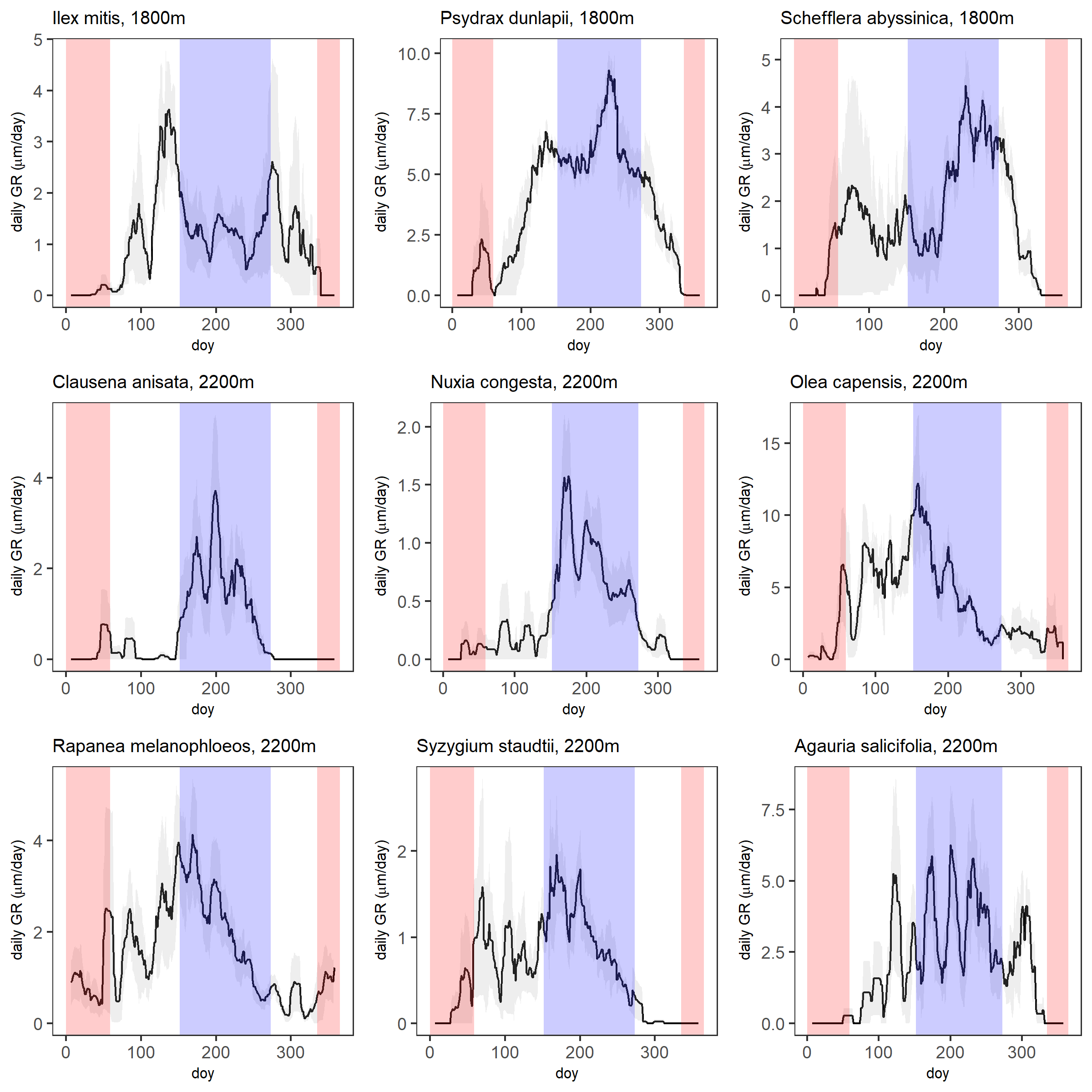


**Figure S3:** Examples of daily growth rate (daily GR) dynamics for selected trees growing in high elevations (1800 and 2200 m a.s.l.). Wet season is depicted as blue area, while dry season in depicted in red.


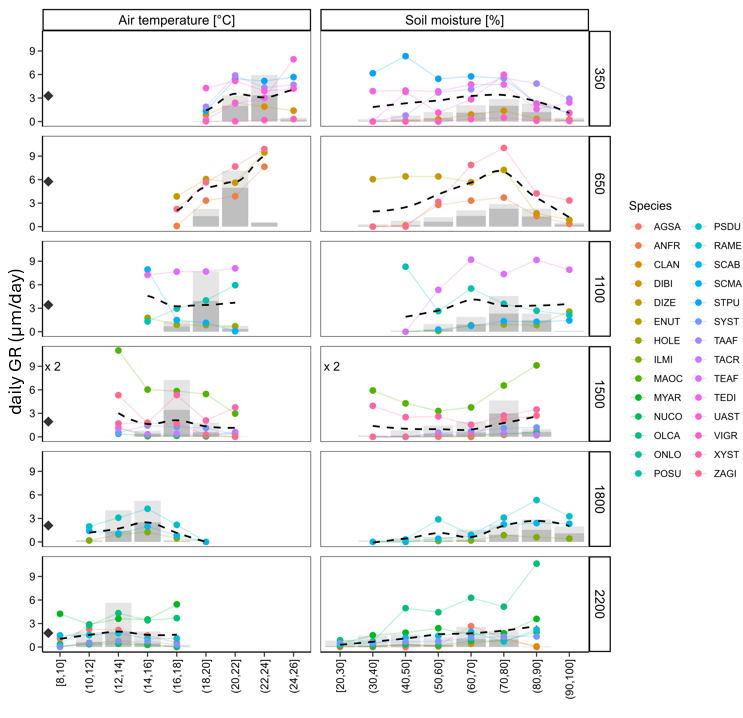


**Figure S4:** Daily growth rates (daily GR, µm/day) across the range of air temperature and soil moisture for trees growing in six elevation belts along the slope of Mount Cameroon. The points and lines represent individual trees and the belt mean is indicated by the dashed line. Grey bars indicate relative frequency of climatic conditions across different ranges in given elevation belt. Bars are divided into darker and lighter parts to indicate the relative frequency of days when hourly GR>0 (darker) and hourly GR=0 (lighter). Diamond next to y-axes highlights long-term mean daily growth rate for a given elevation belt. Note that growth rates of trees from belts 1500 m a.s.l. were divided by 2 to aid comparison between individual panels on the same scale of y-axes.

**
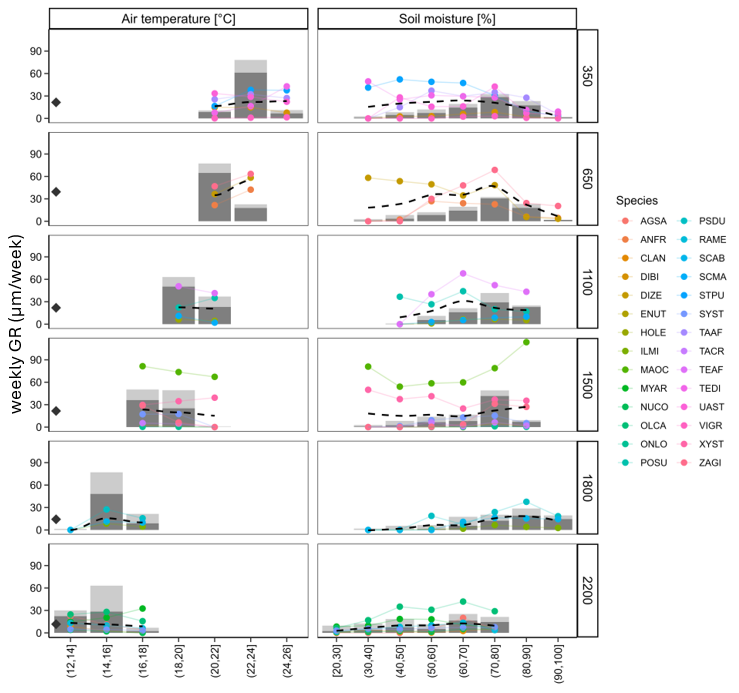
**

**Figure S5:** Weekly growth rates (weekly GR, µm/week) across the range of air temperature and soil moisture for trees growing in six elevation belts along the slope of Mount Cameroon. The points and lines represent individual trees and the belt mean is indicated by the dashed line. Grey bars indicate relative frequency of climatic conditions across different ranges in given elevation belt. Bars are divided into darker and lighter parts to indicate the relative frequency of days when hourly GR>0 (darker) and hourly GR=0 (lighter). Diamond next to y-axes highlights long-term mean daily growth rate for a given elevation belt. Note that growth rates of trees from belts 1500 m a.s.l. were divided by 2 to aid comparison between individual panels on the same scale of y-axes.


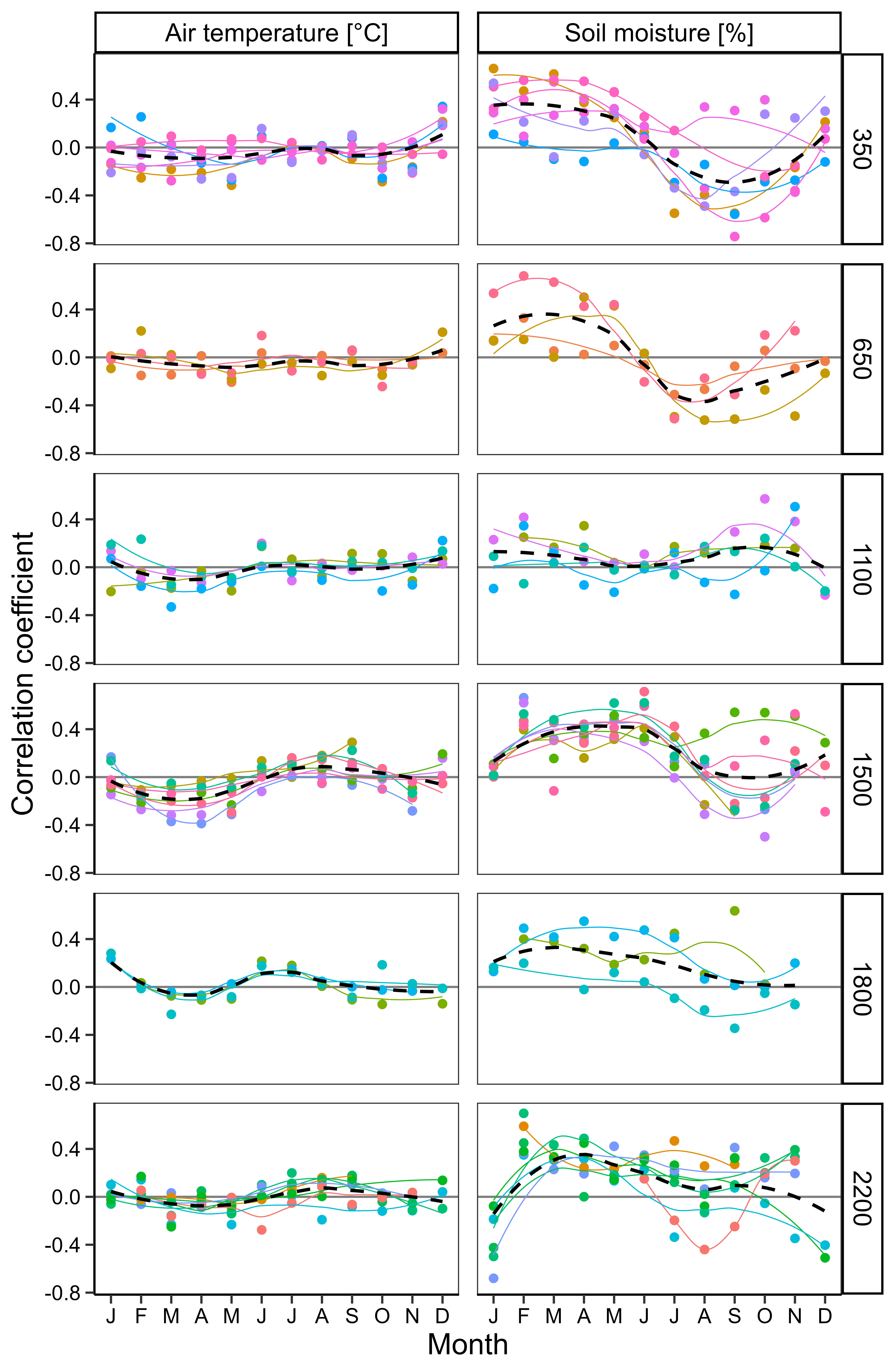


**Figure S6:** Correlation coefficients between climatic variable (air temperature and soil mositure) and daily radial growth rates (daily GR) during specific calendar months for trees growing in six elevation belts along the slope of Mount Cameroon. The points and lines represent individual trees and the belt mean is indicated by the dashed line. For color legend refer to Figure 5 or S4.

**
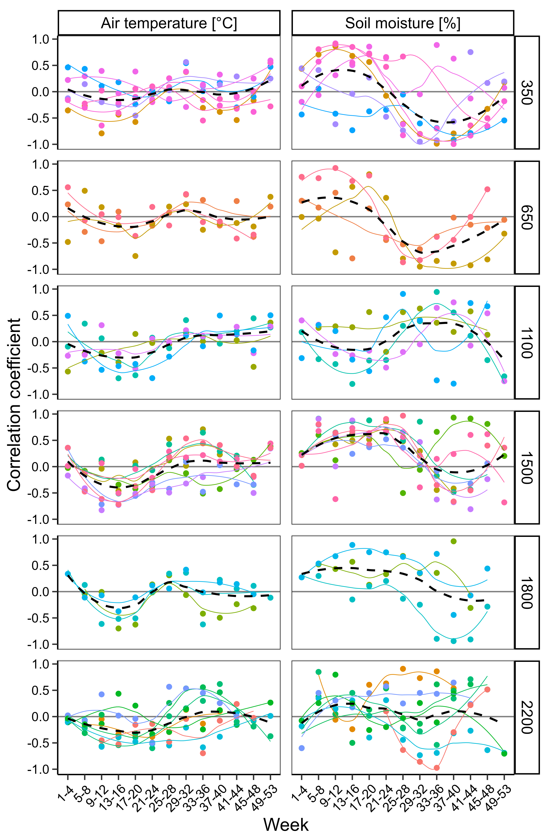
**

**Figure S7:** Correlation coefficients between climatic variable (air temperature and soil mositure) and weekly radial growth rates (weekly GR) during specific calendar weeks for trees growing in six elevation belts along the slope of Mount Cameroon. The points and lines represent individual trees and the belt mean is indicated by the dashed line. For color legend refer to Figure 5 or S4


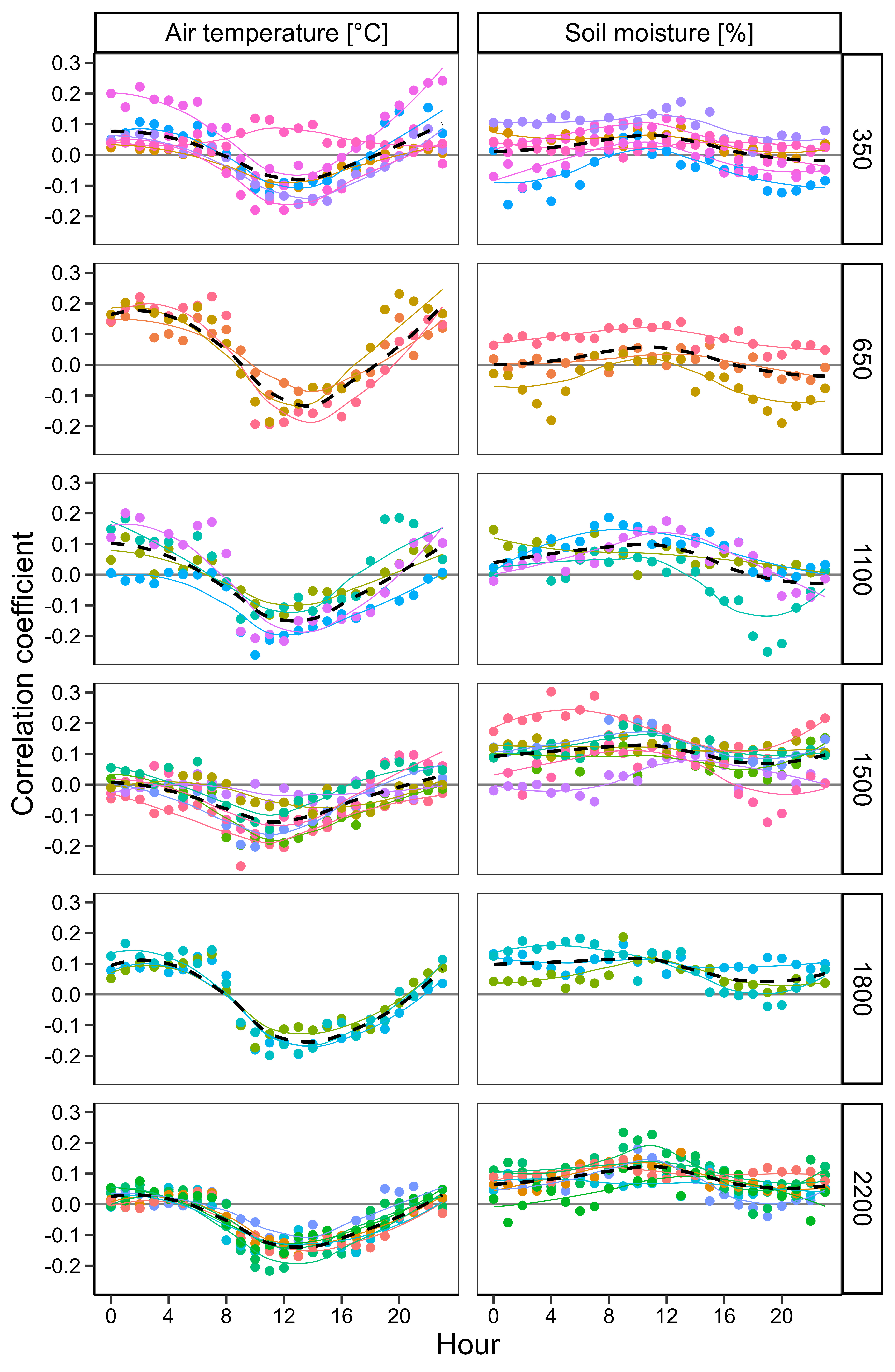


**Figure S8**: Correlation coefficients between climatic variable (air temperature and soil mositure) and radial growth rates during specific hour of the day for trees growing in six elevation belts along the slope of Mount Cameroon. The points and lines represent individual trees and the belt mean is indicated by the dashed line. For color legend refer to Figure 5 or S4.


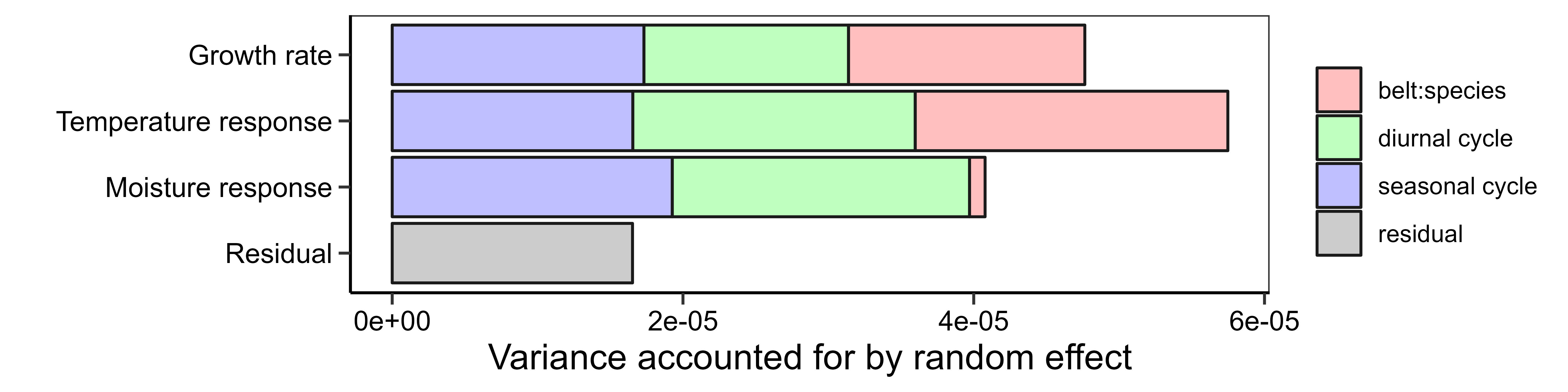


**Figure S9**: Variance in hourly radial growth rate accounted for by random effects of linear mixed-effects model. The model considered variation in growth rate and its response to temperature and soil moisture (y-axes). Tested random predictors include interspecific variability (red), diurnal (green) and seasonal variation (blue).


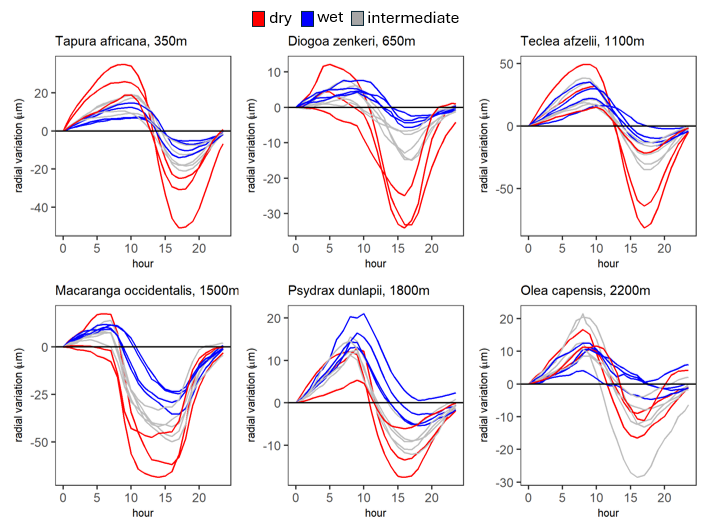


**Figure S10:** Examples of diurnal patterns of trunk shrinking and swelling for selected trees across six elevation belts along the slope of Mount Cameroon. Colors are used to highlight months of dry (red), wet (blue) or intermediate (gray) seasons.


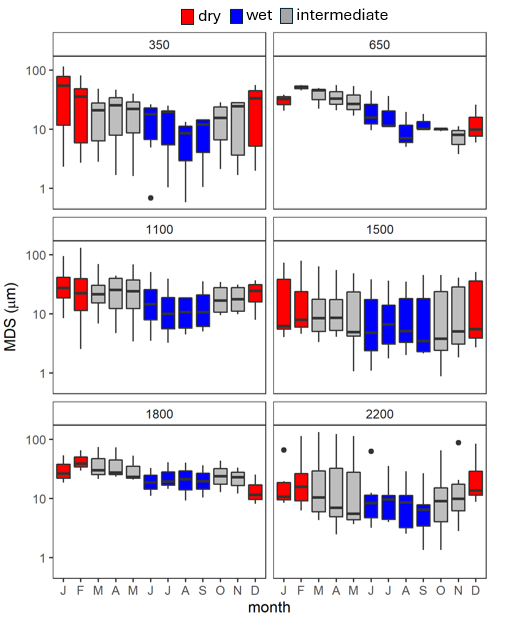


**Figure S11:** Maximal trunk daily shrinkage (MDS) in calendar months for six elevation belts along the slope of Mount Cameroon. Colors are used to highlight months of dry (red), wet (blue) or intermediate (gray) seasons.
